# Does Visual Speed of Processing Training Improve Health-Related Quality of Life in Assisted and Independent Living Communities?: A Randomized Controlled Trial

**DOI:** 10.1093/geroni/igaa029

**Published:** 2020-07-31

**Authors:** Fredric D Wolinsky, Michael P Jones, Megan M Dotson

**Affiliations:** 1 Department of Health, Management and Policy, College of Public Health, The University of Iowa; 2 College of Nursing, The University of Iowa; 3 Department of Biostatistics, College of Public Health, The University of Iowa; 4 Department of Community and Behavioral Health, College of Public Health, The University of Iowa

**Keywords:** Attention control group, Clinical trials, Cognitive training, Health-related quality of life, Senior living communities

## Abstract

**Background and Objectives:**

Visual speed of processing training had clinically and statistically significant beneficial effects on health-related quality of life among 2,802 healthy community-dwelling adults aged 65–94 years at 2 and 5 years post-training in the Advanced Cognitive Training for Independent and Vital Elderly randomized controlled trial. We examined whether that effect would be found among older adults in assisted and independent living communities.

**Research Design and Methods:**

We conducted a two-arm, parallel randomized controlled trial stratified by assisted versus independent settings in 31 senior living communities and enrolled 351 adults aged 55–102 years. The targeted intervention dose was 10 hr at baseline with 4-hr boosters at 5 and 11 months. The intervention group received computerized visual speed of processing training, while the attention control group solved computerized crossword puzzles. The health-related quality of life outcomes were the Short-Form 36-item Health Survey’s mental and physical component *T* scores. Linear mixed-effect models were used.

**Results:**

Visual speed of processing, assisted living, and their interaction had no clinically or statistically significant effects on the physical component *T* scores. However, visual speed of processing (*p* = .022), assisted living (*p* = .022), and their interaction (*p* = .007) had clinically and statistically significant effects on the mental component *T* scores. The estimated marginal means revealed a small effect-sized positive 2.2 point visual speed of processing training effect in the independent living communities, but a clinically important harmful −4.2 point visual speed of processing training effect in the assisted living communities.

**Discussion and Implications:**

Given the medium-sized harmful effect of visual speed of processing training among those in the assisted living communities, caution is advised when using these two visual speed of processing training modalities in assisted living communities until further research verifies or refutes our findings and the underlying etiological pathways.

**Translational Significance:** Improving quality of life in senior living communities is a priority for clients, families, and providers. Visual speed of processing is viable for achieving this goal in studies conducted among community-dwelling older adults. But here we show that those beneficial effects do not extend to assisted living communities.

Normal aging includes declines in multiple cognitive domains such as processing speed, short- and long-term memory, orientation, attention, reasoning, abstract thinking, and perception (1,2). Birren (3) first suggested that age-related linear declines in processing speed (the time it takes to complete mental tasks) during adulthood might be the principal driver of declines in the other cognitive domains. Salthouse (4–8) elaborated and formalized processing speed theory, hypothesizing two ways that it affects other cognitive abilities. First, Salthouse (8) argued that reduced processing speed restricts the time available to successfully accomplish a task when certain other cognitive processes were completed too slowly—the limited time mechanism. Second, Salthouse (8) argued that reduced processing speed increased the loss of early cognitive processing task results through decay or displacement before they were needed for later tasks—the simultaneity mechanism. Considerable evidence supports the claim that processing speed is a principal driver of age-related cognitive decline (9–11).

Salthouse (9) also noted that six different types of processing speed have been considered. These included decision speed (response time to moderately complex cognitive content), perceptual speed (response time to relatively simple cognitive content), psychomotor speed (response time for finger tapping or location drawing stimuli), reaction time (response time for choosing between visual stimuli with keypress methods), psychophysical speed (inspection time for visual or auditory stimuli), and the time course of individual responses (event-related potential). Several indicators for each type of processing speed exist, but the more complex potentially mask the relationship between processing speed and executive function (working memory, cognitive flexibility, and inhibitory control) because both involve goal maintenance, manipulation of information in working memory, and decision-making (12).

Given the diverse types of processing speed, several different cognitive training interventions have been developed. Among the decision speed approaches, the most-studied visual speed of processing training intervention was developed by Ball and Roenker (10,13–15). It was designed to improve driving ability and safety, which are compromised in older adults due to declines in age-related processing speed, peripheral vision and processing, and attentional resources and the ability to ignore distractions. This training involves improving the exposure time needed to (a) identify which of two target objects are presented visually, (b) locate a second simultaneously presented target object, and (c) locate the second target in the presence of distractors. The standard measure of visual speed of processing is the Useful Field of View test (15), which has subtests that tap processing speed, divided attention, and selective attention.

Visual speed of processing training was used as one of three interventions in the multisite Advanced Cognitive Training for Independent and Vital Elderly (ACTIVE) randomized controlled trial (RCT) (16–19) that included 2,802 healthy community-dwelling adults aged 65 years and older. The three cognitive training interventions—episodic memory, inductive reasoning, and visual speed of processing—affected their targeted proximal and primary outcomes over 2- to 10-year follow-ups (16–19). Only visual speed of processing training, however, statistically and clinically affected distal health outcomes like health-related quality of life, depression, self-rated health, and internal locus of control for up to 5 years (20–26).

In this article, we examined whether visual speed of processing training had a beneficial effect on health-related quality of life in the MOOD Study (27,28), an RCT conducted among older adults in senior living communities. The MOOD Study’s three aims were to examine whether visual speed of processing training improved processing speed (28), reduced depressive symptoms and depression (29), and improved health-related quality of life. Our examination of the effects of visual speed of processing training on health-related quality of life is important because since 2007, more than 700 senior residential facilities and communities have installed some type of computerized cognitive training programs, and 300 of these used a single program (Brain Fitness, https://www.dakim.com/dakim/) that has never been tested in these communities (30,31).

Furthermore, to our knowledge, visual speed of processing training has never been studied in senior living communities, where the average age is higher, and residents generally have substantially more comorbidities and poorer general health than community-dwelling adults (32). For example, in ACTIVE, the average age was 73.6 years, 15.7% self-rated their health as either fair or poor, and only those with minimal difficulties in three activities of daily living (bathing, hygiene, and dressing) were allowed to enroll (16–19). National data from assisted living communities, however, show that the average age was 86.9 years, 21.7% self-rated their health as fair or poor, and 64% had limitations in at least one activity of daily living (64% with bathing, 57% with walking, 48% with dressing, and 40% with toileting). Moreover, in assisted living, 52% had high blood pressure, 42% had arthritis, 42% had Alzheimer’s disease or a related dementia, 34% had heart disease, and 31% were depressed (32–34). These age, self-rated health, and comorbidity differences could result in visual speed of processing training being somewhat less effective for those in assisted living, especially if maximal thresholds on these characteristics existed above which the intervention would not be effective.

Unlike assisted living communities that are regulated by the federal government, independent living communities are regulated at the state level and therefore national data on them are sparse. That said, older adults in independent living communities are generally self-sufficient, require no assistance with activities of daily living, and have no need for 24/7 nursing or medical care. They generally purchase their own maintenance-free living setting (condo, townhouse, or patio home) in a secure senior living community, and enjoy a variety of amenities including onsite restaurants, recreational facilities, and social activities. Accordingly, there is the possibility that visual speed of processing training might be more effective in independent versus assisted living communities (35). Therefore, including participants from both the assisted and independent living settings in 31 senior living communities, the MOOD Study permits direct testing for heterogeneity of treatment effects between these settings.

## Method

### Ethics and Approvals

All MOOD Study personnel received, completed, and passed human subjects training and testing as well as continuing education using the Collaborative Institutional Training Initiative programs (https://about.citiprogram.org/en/homepage/). Independent Review Board approval was obtained from the University of Iowa (UI; Protocol 201208786) on September 18, 2012, with continuing and modification reviews approved thereafter. The study protocol was registered with ClinicalTrials.gov (NCT 01763216) on January 3, 2013. The first individual participant was enrolled on May 17, 2013, and the last on October 22, 2015. All follow-up interviews were completed by October 12, 2016.

### Settings

We identified assisted living communities in eastern Iowa using state registries and web searches. Assisted living community directors were then asked about their potential interest in participating. Site visits for those expressing interest were conducted that included an overview of the research project, the roles and responsibilities of both the assisted living and UI project staff, and a facility tour to determine appropriate locations for the placement of training stations. If the assisted living community directors wanted to proceed, memoranda of agreement were signed.

We tailored the protocol delivery logistics (but not the intervention itself) to best fit each setting. For example, in some of the assisted living communities, a separate room was selected to house both of the project training stations, while in others the training stations were placed in separate rooms or common areas. Similarly, in some of the assisted living communities, there was only one study liaison, while in others there were two or more. Honoraria of $5,000 were provided to each assisted living community to offset their costs of study participation, including internet access, staff participation, and training station furnishings. Selected senior living community staff were designated as Study Liaisons and received, completed, and passed human subjects training and testing using the Collaborative Institutional Training Initiative programs. UI research team members then showed them how to properly recruit, informally assess cognitive capacity to consent based on the participant’s ability to explain back the basic nature of the study, obtain written consent, and then how to enroll and teach participants to use the assigned computerized software. These training sessions lasted about two days.

The MOOD Study protocol originally targeted the recruitment of 10 participants from each of up to 30 assisted living communities. This was quite optimistic given that there were only about 250 of these communities throughout Iowa, nearly half of which had fewer than 11 beds, and with logistical constraints limiting our ability to go beyond the eastern half of the state. Furthermore, early on the willingness for the assisted living community directors to express interest in having site visits conducted was less than expected. Anecdotal feedback suggested that this was likely due to the hesitation to participate in research projects due to the potential disruption to patient and staff routines. Therefore, eligibility was extended to the coresiding independent living settings at participating assisted living communities.

### Eligibility and Interviewing

The original inclusion criteria targeted those who were 60 years old or older (which was the minimum age eligibility criteria for state-subsidized assisted living communities in Iowa), the ability to sign meaningful informed consent based on the Study Liaison’s informal assessment of participant’s cognitive function, and a demonstration of physical acuity to use a monitor, keyboard, and mouse. Because seven of the participating assisted living communities used participants aged 55 years or older as their lower age boundary for admission, we reduced the minimum age to 55. No further changes to any sampling or eligibility criteria were made.

Baseline telephone interviews averaged 35 min and were conducted by UI research assistants using REDCap (36), with follow-up telephone interviews conducted at 5–8 weeks (posttraining), and at 6 and 12 months that averaged 30 min. UI research assistants were blinded for all interviews. Individual participants received $25 for each of their completed interviews. Study Liaisons administered the computerized Useful Field of View (15,37) tests after each telephone interview.

### Randomization

The study biostatistician (M. P. Jones) wrote a computer program to generate randomization assignment letters. Sets of randomization assignment letters were generated separately for each assisted and independent living setting within each of the 31 senior living communities. The allocation ratio was one-to-one and permuted block sizes of two and four were used. Randomization assignment letters were placed in sequentially numbered, sealed opaque envelopes within each assisted and independent living setting within each of the 31 senior living communities. The randomization assignment letters were secured in the project coordinator’s (M. M. Dotson) office, and only after a participant’s baseline interview and Useful Field of View test (37) were completed did she open the next sequential letter for that community’s assisted or independent living setting.

### Intervention

We used two second-generation versions of the visual speed of processing training that was used in ACTIVE (16–19; *Road Tour/Double Decision*, https://www.brainhq.com/why-brainhq/). Both have been shown to be equally effective on processing speed (28). They, however, differed from the ACTIVE platform in several ways including the use of graphic user interfaces operating in Windows rather than MS-DOS, the addition of gaming elements (in *Road Tour*), the elimination of the need for standby supervision, and the suitability for individual delivery. It is also important to note that in ACTIVE all training was delivered in small groups of three to five participants, immediately after which the group trainer led discussions lasting about 20 min emphasizing speed of processing’s importance and effects on other everyday life activities. No such discussions occurred in the individual self-training sessions in the MOOD Study.

In the MOOD Study, the compact disk platform *Road Tour* was used until Posit Science rebranded and replaced it with *Double Decision* on the internet. At the lowest challenge level, intervention participants saw a car or truck (the object) in the center of the monitor with eight locations (moons) in the same near-periphery orbit around it. One of those locations held the route 66 road sign (target), while the other seven held rabbit signs (distractors). The goal was to correctly identify the object and the target location as quickly as possible (measured in milliseconds). The platforms used a brief assessment of the individual participant to set the initial challenge levels, which were adaptively increased by adding more distractor signs in more distal peripheral orbits, allowing the target to appear in any location on the monitor, morphing the objects to become more similar, and increasing the complexity of the background images. But these changes occurred only after correct identification of the object (car or truck) and target location (route 66 sign) in ≥75% of the trials at the prior complexity level. The targeted training dose was 10 hr within 5–6 weeks of baseline, with four additional hours at 5 and 11 months, corresponding to the ACTIVE booster strategy. Time spent on task was electronically recorded by the visual speed of processing training platforms.

### Attention Control

We used Boatload Puzzles, LLC’s Boatload of Crosswords (https://www.boatloadpuzzles.com/) for the attention control participants. They saw a traditional puzzle format but used the mouse and keyboard to enter answers to the row and column clues. Individual participants could choose the size and complexity of the puzzle, and use radio buttons to fill in letters or words, show incorrect entries in a different colored font, and/or solve the entire puzzle. The same training schedule was used for the attention control participants. Boatload of Crosswords, however, was neither adaptive nor did it electronically record time on task.

### Health-Related Quality of Life

The Short-Form 36-item Health Survey, version 2.0, which is the most widely used patient-reported outcome measure in clinical trials (38), was our outcome measure. As specified in the MOOD Study protocol (NCT 01763216), the physical and mental component *T* scores (PCS-T and MCS-T; which range from 0 for worst health to 100 for best health) were the main outcomes because of their greater accuracy, minimal floor and ceiling effects, enhanced responsiveness, and higher reliability than the eight individual subscale scores (27). Although national norms for older adults were available, they were based on community-dwelling adults in good health, community-dwelling patients in general practice, or members of Medicare Advantage Organizations (39–41). Thus, no appropriate national norms were available for our target population. Moreover, some have suggested that the Short-Form 36-item Health Survey may be problematic in congregate living settings (42,43), and that nationally normed scores may lead to misinterpretations (44).

Accordingly, we calculated the Short-Form 36-item Health Survey physical and mental component *T* scores locally. First, individual items were recoded as recommended, exploratory factor analyses were performed for the items belonging to each subscale, and internal consistency reliability coefficients were estimated. The separate exploratory factor analyses of the items belonging to each subscale revealed single, simple factor structures with all factor loadings ≥0.45, and all coefficient alphas ≥0.75. The eight raw subscale scores were then constructed and analyzed using exploratory factor analysis with orthogonal rotation. The resulting two-factor solution was consistent with expectations, with factor loadings ≥0.59, and explained 68.0% of the variance in the eight subscales. Each of the resulting factor loadings were then multiplied by the individuals’ appropriate raw subscale scores and summed to generate the physical and mental component scores, which were then converted to *T* scores (mean = 50, *SD* = 10) based on sample distribution characteristics. For older adults in RCTs, group-level changes (or difference-in-differences) of ± 2.0, ± 5.0, and ± 8.0 points on the physical and mental component *T* scores are considered to be small, medium, and large clinically important differences (45,46).

### Covariates

The baseline value of the Short-Form 36-item Health Survey physical or mental component *T* score was included as a covariate to focus on changes over 1 year (47). A main effect for assisted versus independent living was included to gauge differences due to residential settings, as was an interaction term of residential setting with visual speed of processing training to test for heterogeneity of treatment effects. Three sets of additional covariates were selected to ensure proper attribution of any observed effects of the main effects for visual speed of processing training and residential setting, or their interaction. The justification for the first covariate set was that we expected those in assisted versus independent living communities would differ in terms of their baseline processing speed. Therefore, the Useful Field of View test (15,37) was used as a covariate. Its three subtests tap processing speed, divided attention, and selective attention are scored from 17 to 500 ms. As with the visual speed of processing training platforms, processing speed reflects the exposure time needed to identify the target object (car or truck), divided attention reflects the exposure time required to colocate a second target (route 66 sign) that is simultaneously presented, and selective attention reflects the exposure time required to colocate the second target in the presence of distractors (rabbit signs). The Useful Field of View composite score is the simple sum of the three subtests and ranges from 51 ms (fastest) to 1,500 ms (slowest). The psychometric properties of this and the other covariate summary scales are shown in Supplementary Table 1.

The justification for the second set of covariates was that community-dwelling older adults have different sociodemographic and health characteristics than those in senior living communities, and that within senior living communities those in assisted versus independent living have different sociodemographic and health characteristics (32–35). Therefore, we included age (years), sex (1 = male, 0 = female), and education (1 = none through 8 = graduate training) as covariates to adjust for sociodemographic factors. The number of comorbid conditions (0–17) was included as a covariate to adjust for health status.

Finally, the justification for the third set of covariates was that community-dwelling older adults have different psychosocial factors than those in senior living communities, and that within senior living communities those in assisted versus independent living have different psychosocial factors (32–35). Accordingly, we included depression, anxiety, and social support to adjust for these psychosocial differences. Depression was measured using the nine-item Patient Health Questionnaire (48) which is routinely used in research and clinical practice to quantify depression and reflects all five of the diagnostic criteria for major depression. Each item has four response options ranging from 0 = not at all, to 3 = nearly every day, for a scale score range of 0–27. The depression score is the simple sum of the nine items. The seven-item Generalized Anxiety Disorder scale (49) was used to measure anxiety. Each item has the same four response options as the depression measure, for a scale score range of 0–21. The anxiety score is the simple sum of the seven items. Social support was measured using five items (having a confidante, someone to turn to, someone to love, someone to get together with for relaxation, and someone to do something enjoyable with) from the Medical Outcomes Study social support scale (50). Each item has five response options ranging from 0 = none of the time, to 4 = all of the time. The social support score is the simple sum of the five items and ranges from 0 to 20.

### Hypotheses

We first hypothesized that compared to those in the crossword puzzles attention control group, those in the visual speed of processing training group would have improved scores on both the Short-Form 36-item Health Survey physical component and mental component *T* scores. The focus on the physical and mental component *T* scores was due to their greater accuracy, minimal floor and ceiling effects, enhanced responsiveness, and higher reliability than the eight subscale scores (27). This first hypothesis was based on prior work that had shown statistically and clinically relevant effects on four or more of the eight Short-Form 36-item Health Survey subscale scores, indicating a broad pattern of effects on the various components of health-related quality of life (21,22). That said, the second hypothesis was that the mental component *T* scores would be more affected than the physical component *T* scores. This second hypothesis was based on the fact that, as a cognitive training intervention, visual speed of processing training should have more immediate and stronger effects on mental health functions, compared to more of a lagged and weaker effect on physical health functions. Moreover, data from our prior work (not shown) were consistent with this expectation (21,22). Finally, our third hypothesis was that despite the known differences in age, health, and well-being described above between community-living older adults and those in senior living communities, as well as between those in assisted versus independent living (12), the visual speed of processing training effects could be the same for both settings. This was based on prior reports of the effect of visual speed of processing training on health-related quality of life from ACTIVE (16–19), which failed to reveal any age or comorbid heterogeneity of treatment effects.

### Analyses

The visual speed of processing training versus attention control groups and the assisted versus independent living communities were compared on the covariates at baseline, and on the baseline and one-year outcomes using chi-squared and Student’s *t* tests. To examine the potential for attrition bias, we used binomial logistic regression analysis to contrast those with complete data at baseline and 1 year versus those without. Next, seven progressively more complex intention-to-treat linear mixed-effects models were estimated, incorporating random effects to account for the clustering within the 31 senior living communities. The first model reflected the traditional RCT equivalence expectation (any observed differences between the two treatment groups were due to chance) and included only a main effect for visual speed of processing training and a random effect to adjust for clustering within sites as well as any recruitment or implementation differences across sites. Model 2 added the baseline outcome *T* score to estimate average visual speed of processing training effects on changes in health-related quality of life over 1 year and to adjust for any differences in baseline scores. Model 3 added a main effect for assisted living status at baseline. Model 4 added the interaction between the visual speed of processing training and assisted living main effects to test for heterogeneity of treatment by residential setting. Model 5 added baseline age, sex, education, and comorbidity to adjust for sociodemographic factors and illness burden. Model 6 added the baseline Useful Field of View test to adjust for any initial differences in processing speed. Finally, model 7 added the baseline depression, anxiety, and social support scores to adjust for any psychosocial differences at baseline. All analyses were conducted using IBM SPSS Software, v25 and v26.

## Results

### Descriptive

Supplementary Figure 1 contains the CONSORT (Consolidated Standards of Reporting Trials) flow chart (28,29). A total of 370 participants were consented, of whom 19 did not meet the inclusion criteria and were not randomized. The 351 randomized participants were recruited from 31 senior living communities, with a mean of 11.3 participants per site. Complete data at baseline and at the 1-year follow-up were available for 300 (85.5%) of the 351 participants. The mean visual speed of processing training treatment dose by study completion was 9.8 hr (interquartile range = 4.9–14.7 hr) and did not significantly differ between the assisted versus independent living communities (8.9 vs 10.5 hr, *p* = .109).

Table 1 contains the unadjusted baseline means or percentages for the covariates and the Short-Form 36-item Health Survey physical and mental component *T* scores at 1 year. Overall, the mean age was 80.7 years, 72.3% were women, and 42.3% resided in assisted living. A baseline comparison of the two treatment groups (the next to the last column) found significant differences only for the baseline physical component *T* scores, with those in the visual speed of processing training group having lower scores than those in the attention control group. The four-group treatment-by-community comparisons (the last column) among the covariates indicated that at baseline there were significantly more men in the assisted living communities, that visual speed of processing training participants in the assisted living communities had lower education levels than their counterparts in the attention control group, that depression and anxiety scores were highest and social support scores were the lowest in the assisted living community visual speed of processing training group, and that processing speed was slowest in the assisted living community attention control group. Among the baseline outcomes, those in the assisted living communities who received the visual speed of processing training had the lowest physical component *T* scores. At the 1 year assessment, those in the assisted living communities who received visual speed of processing training had the lowest physical and mental component *T* scores.

**Table 1.** Unadjusted Means or Percentages for the Covariates and 36-Item Health Survey Physical and Mental Component *T* Scores for the 300 Participants With Complete Baseline and 1-Year Follow-Up Data by Treatment Group and Living Arrangement

	Visual speed of processing treatment			Crossword puzzles attention control				
Measure	Assisted living, *N* = 64	Independent living, *N* = 84	Total, *N* = 148	Assisted living, *N* = 63	Independent living, *N* = 89	Total, *N* = 152	Two-group *p* value^a^	Four-group *p* value^b^
Baseline covariates								
Age (years)	81.2	79.7	80.3	82.4	80.1	81.0	.501	.268
Percent male	35.9	17.9	25.7	34.9	25.8	29.6	.449	.045
Education level (1–8)^c^	5.4	6.0	5.7	5.8	6.1	6.0	.185	.036
Comorbidity count (0–17)	5.8	4.8	5.3	5.2	4.7	4.9	.261	.059
UFOV composite (ms)^d^	596.8	455.2	516.8	664.2	421.5	521.2	.901	.001
Depression^e^	5.4	3.2	4.2	4.1	3.7	3.9	.511	.005
Anxiety^f^	3.5	2.0	2.7	2.1	2.1	2.1	.121	.015
Social support^g^	13.4	14.9	14.1	13.1	14.4	14.3	.466	.045
Baseline health-related quality of life^h^								
Physical component *T* scores	45.5	51.3	48.8	49.3	52.5	51.2	.042	.001
Mental component *T* scores	49.0	51.8	50.6	50.1	48.9	49.4	.319	.216
1*-*year health-related quality of life^h^								
Physical component *T* scores	44.8	49.9	47.7	48.4	49.6	49.1	.234	.011
Mental component *T* scores	46.2	52.1	49.6	49.8	49.6	49.0	.659	.013

*Notes*: UFOV = Useful Field of View.

^a^Combined visual speed of processing training vs combined crossword puzzles attention control groups. ^b^Visual speed of processing training in assisted living vs visual speed of processing training in independent living vs crossword puzzles attention control groups in assisted living vs crossword puzzles attention control groups in independent living. ^c^Educational level was coded 1 = none, 2 = grades 1–8, 3 = grades 9–11, 4 = high school or general education development completion, 5 = vocational or trade school, 6 = some college, 7 = college graduate, and 8 = graduate training. ^d^UFOV composite scores range from 51 to 1,500 ms. ^e^Depression scores range from no depression (0) to maximal depression (27). ^f^Anxiety scores range from no anxiety (0) to maximal anxiety (21). ^g^Social support scores range from no support (0) to maximal support (20). ^h^Physical component *T* scores and mental component *T* scores range from 0 to 100 with a mean of 50 and a *SD* of 10.

### Potential Attrition Bias

Binomial logistic regression contrasted having versus not having complete data at both baseline and 1 year. The baseline values of treatment assignment group, assisted living, age, sex, education, comorbidity, Useful Field of View, depression, anxiety, and social support were included as predictors (data not shown). Only assisted living (adjusted odds ratio [AOR] = 0.335, *p* = .005) and the Useful Field of View (AOR = 0.998, *p* = .001) were significant predictors, indicating that those in the assisted living communities and those having slower processing speed were less likely to have complete data at 1 year. Because there was no indication of heteroscedastic error (Hosmer–Lemeshow = 7.34, *p* = .501), the model fit the data well (area under the curve = 0.767), and both significant predictor variables were included as covariates in the final analysis (Model 7), the potential for attrition bias was minimized.

### Linear Mixed Effect Models

Tables 2 and 3 contain the main effect regression coefficients (*b*_*s*_) for visual speed of processing training and assisted living as well as their interaction from the complete case intention-to-treat analyses of the Short-Form 36-item Health Survey physical and mental component *T* scores, respectively. There were no statistically significant effects (*b*_*s*_) on the physical component *T* scores. However, significant visual speed of processing training (*p* = .033), assisted living (*p* = .013), and their interaction (*p* = .014) effects (*b*_*s*_) were observed on the mental component *T* scores once the interaction term was introduced in Model 4. These effects remained significant as the baseline covariates were progressively added. In the final model (M_7_), visual speed of processing training (*p* = .022), assisted living (*p* = .022), and their interaction (*p* = .007) all remained statistically significant. Moreover, their *b*_*s*_ remained comparable.

**Table 2.** The Effects of Visual Speed of Processing Training, Assisted Living, and Their Interaction Obtained From Seven Progressive Intention-to-Treat, Random Effects Linear Mixed Models for the 36-Item Health Survey Physical Component *T* Scores at 1 Year

	Visual speed of processing training		Assisted living		Their interaction	
Models (M_n_)	*b*	*p*	*b*	*p*	*b*	*p*
M_1:_ Equivalance expectation with a random effect for site clustering	1.410	.229				
M_2:_ M_1_ + Baseline physical component *T* score main effect	−0.415	.582				
M_3:_ M_2_ + Baseline assisted living main effect	−0.502	.504	−1.932	.060		
M_4:_ M_3_ + Visual speed of processing training with assisted living interaction effect	0.845	.473	−0.753	.561	−2.266	.139
M_5:_ M_4_ + Baseline age, sex, education, and comorbidity main effects	0.719	.541	−1.459	.272	−2.135	.161
M_6:_ M_5_ + Baseline useful field of view composite score main effect	0.775	.514	−1.255	.353	−2.314	.133
M_7:_ M_6_ + Baseline depression, anxiety, and social support score main effects	1.605	.212	−0.636	.659	−3.118	.059

**Table 3.** The Effects of Visual Speed of Processing Training, Assisted Living, and Their Interaction Obtained From Seven Progressive Intention-to-Treat, Random Effects Linear Mixed Models for the 36-Item Health Survey Mental Component *T* Scores at 1 Year

	Visual speed of processing training		Assisted living		Their interaction	
Models (M_n_)	*b*	*p*	*b*	*p*	*b*	*p*
M_1:_ Equivalance expectation with a random effect for site clustering	−0.700	.583				
M_2:_ M_1_ + Baseline mental component *T* score main effect	0.381	.726				
M_3:_ M_2_ + Baseline assisted living main effect	0.417	.702	1.747	.224		
M_4:_ M_3_ + Visual speed of processing training with assisted living interaction effect	3.618	.033	4.518	.013	−5.445	.014
M_5:_ M_4_ + Baseline age, sex, education, and comorbidity main effects	3.796	.027	5.392	.004	−5.519	.013
M_6:_ M_5_ + Baseline useful field of view composite score main effect	4.294	.011	5.112	.007	−6.440	.004
M_7:_ M_6_ + Baseline depression, anxiety, and social support scores	4.188	.022	4.610	.022	−6.352	.007

Supplementary Table 2 contains the estimated marginal means for the mental component *T* scores for Models 4–7, which revealed a classic heterogeneity of treatment effects pattern. According to the final model (M_7_), in the assisted living communities the visual speed of processing training group had a lower mental component *T* score (46.7) than the attention control group (50.9) for a difference of −4.2 points. In contrast, in the independent living communities, the visual speed of processing training group had a higher mental component *T* score (51.3) than the attention control group (49.1) for a difference of 2.2 points. This indicated a 6.4 point difference-in-differences (overall point gap) between the visual speed of processing training effects in the assisted versus independent living communities, reflecting a medium-sized clinically important difference (40,41).

### Sensitivity Analyses

Given the unexpected heterogeneity of treatment effect, seven ad-hoc sensitivity analyses were conducted, all of which had reduced statistical power due to sample restrictions. Therefore, the focus was on the observed difference-in-differences in the mental component *T* scores rather than the *p*-values. The first sensitivity analysis reestimated Model 7 separately for those in the assisted versus independent living communities to clarify the interpretation of the interaction effect. Those results (data not shown) revealed a difference-in-differences of 5.3 points, comparable to that shown in Table 3 and Supplementary Table 2.

The second sensitivity analysis explored whether a mental component *T* score threshold existed above (better than) which a beneficial visual speed of processing training effect would be observed. This was plausible given the small but clinically important 2.8 *T* score points lower (worse) baseline mental component mean for those in the visual speed of processing training assisted versus independent living groups. For this nonintention-to-treat analysis the sample was restricted to the 200 participants (77 in assisted versus 123 in independent living communities) having mental component *T* scores at or above (faster than) the overall *T* score mean of 50.0 points. These results were comparable to those from Model 7 shown in Table 3 and Supplementary Table 2 and reflected a difference-in-differences of 6.3 points on the mental component *T* scores.

The third sensitivity analysis explored whether a Useful Field of View (37) threshold existed below (faster than) which a beneficial visual speed of processing training effect would be observed. This was plausible given the significantly (192.2 ms) higher (slower) baseline mean for those in the assisted versus independent living communities. For this nonintention-to-treat analysis the sample was restricted to the 147 participants (39 in assisted vs 108 in independent living communities) having Useful Field of View scores at or below (faster than) the mean (113.3 ms) in the independent living communities. These results were also comparable to those from Model 7 shown in Table 3 and Supplementary Table 2 and reflected a difference-in-differences of 8.8 points on the mental component *T* scores.

A fourth sensitivity analysis focused on whether a training time threshold existed that might account for the observed heterogeneity of treatment effect. This was possible because those who received visual speed of processing training in the assisted living group wound up having fewer hours on task than those in the independent living group (8.9 vs 10.5 hr, *p* = .109). Because training time was not available for those in the crossword puzzle groups, this non-intention-to-treat analysis was restricted to visual speed of processing training participants who received at least 8 hr of total training, a threshold previously used in ACTIVE (16–19). The results of this analysis (data not shown) were yet again comparable to those shown in Model 7 of Table 3 and Supplementary Table 2 and reflected a difference-in-differences of 8.8 points on the mental component *T* scores.

The fifth and sixth sensitivity analyses explored whether depression and anxiety thresholds existed below (better than) which the visual speed of processing training effect would be beneficial. For these non-intention-to-treat analyses, the samples were restricted to (a) the 155 participants (53 in assisted vs 102 in independent living communities) having depression scores at or below (less than) the mean (3.5 points) in the independent living communities, and (b) the 186 participants (65 in assisted vs 121 in independent living communities) having anxiety scores at or below (less than) the mean (2.1 points) in the independent living communities. The results (data not shown) for the depression and anxiety sensitivity analyses revealed difference-in-differences of 5.1 and 6.3 points on the mental component *T* scores, both of which were also comparable to that shown in Table 3 and Supplementary Table 2.

The last sensitivity analysis was restricted to those in the assisted living communities who received visual speed of processing training. Those 62 participants were separated into two groups—those whose Short-Form 36-item Health Survey mental component *T* scores improved versus those that declined. This analysis explored whether differences between the improvers and decliners were related to any of the covariates. The mean change from baseline to 1 year among those who improved was −6.4 ms while the mean change among those who declined was +11.1 ms (*p* < .001). The only significant unadjusted differences between these groups on the baseline covariates (data not shown) were that the improvers were more educated (*p* = .031) and had lower mental component *T* scores (*p* = .028) at baseline. Logistic regression using the baseline covariates to model improvers versus decliners revealed that only the mental component *T* scores were significant (AOR = 0.929, *p* = .031), indicating a modest, routine ceiling effect such that it was easier for those with lower mental component *T* scores to improve.

## Discussion

In this article, we evaluated whether visual speed of processing training, when used with older adults in assisted and independent living settings in senior living communities (28,29), would have similar beneficial effects on health-related quality of life as those reported for community-dwelling older adults (21,22,25). We hypothesized that visual speed of processing training would have these beneficial effects for several reasons. First, we used two second-generation versions (*Road Tour/Double Decision*) of the computerized training platform used in ACTIVE (16–19). While many other computerized “brain games” were commercially available, the efficacies for most of them have not been demonstrated (50). In contrast, the visual speed of processing training platforms used here have been the most studied (50). This includes their use in the three largest cognitive training RCTs ever conducted (16–19,51–53). Based on those studies, these visual speed of processing training platforms received PEDro (Physiotherapy Evidence Database; www.pedro.org.au) scores of 9 out of a possible 10 (54). PEDro scores are based on the Delphi List for quality assessments of RCTs (55), which are consistent with CONSORT evaluation guidelines (56), resulting in Level I (the highest) rating in the evidence hierarchy (51).

Second, these visual speed of processing training platforms were the only ones that met all of the criteria for brain training games established by the National Academy of Medicine (57), as well as the five requirements for successful training (58). Finally, prior reports of the effect of visual speed of processing training on health-related quality of life from ACTIVE (16–19) did not reveal any age or comorbidity heterogeneity of treatment effects. Therefore, we expected that the visual speed of processing training effects on health-related quality of life would be the same in both the assisted versus independent living communities that we studied regardless of the known differences between community-living older adults and those in senior living communities, as well as between those in assisted versus independent living (12).

But that was not what we found. Our analyses revealed that there were no significant effects of visual speed of processing training on the Short-Form 36-item Health Survey (38) physical component *T* scores (Table 2). Clinically and statistically significant effects of visual speed of processing training, assisted living, and their interaction were observed, however, on the mental component *T* scores (Table 3, Models 4–7). The marginal means from the final model (M_7_ in Supplementary Table 2) for the mental component *T* scores estimated from the linear mixed effect models for the assisted living communities were 46.7 for the visual speed of processing training group versus 50.9 for the attention control group, whereas for the independent living communities the marginal means were 51.3 for the visual speed of processing training group versus 49.1 for the attention control group. This represents a clinically important −4.2 point visual speed of processing training effect for those in assisted living versus a 2.2 point visual speed of processing training effect for those in independent living, for an overall medium-sized (6.4 points) clinically important difference-in-differences (45,46). Furthermore, this harmful heterogeneity of treatment effect for the assisted living communities was robust in stratified analyses (assisted vs independent living communities), across five different sensitivity analyses checking for mental component *T* score, Useful Field of View (37), training time, depression (48), and anxiety (49) thresholds, as well as differences in personal characteristics between those in assisted living who improved versus declined on the mental component *T* scores.

The null findings on the Short-Form 36-item Health Survey physical component *T* scores were consistent with the modest evidence of the distal transfer of cognitive training in adequately powered RCTs (59,60), especially to physical performance related outcomes. The modest beneficial visual speed of processing training effects on the mental component *T* scores in the independent living communities versus its substantially harmful effects in the assisted living communities, however, were unexpected. This is surprising in light of the significant and comparable Useful Field of View gains (no heterogeneity of treatment effects) previously demonstrated (28). Moreover, the non-intention-to-treat sensitivity analyses revealed that this was not due to mental component *T* score, Useful Field of View (37), training time, depression (48), or anxiety (49) thresholds. Thus, further research is needed to confirm these unexpected results in other visual speed of processing training RCTs among older adults in senior living communities.

### A Potentially Plausible Conceptual Explanation

An explanation of our findings might be found in Selye’s (61–64) three-stage general adaptation syndrome (alarm, resistance, and exhaustion). He argued that the reaction to an *acute* stressful situation results in a fight or flight response. This leads to an immediate spike in cortisol production, followed by a return to normal cortisol levels once the *acute* stressor has passed. The stress process, however, operates differently in the presence of *chronic* stressors. Recurrent exposure to uncontrollable social evaluative threats (negative self-identity perceptions) also results in elevated cortisol levels, but cortisol recovery is inhibited over time, leading to negative emotional outcomes (65,66). Negative reactive effects to *chronic* stress also lead to reductions in cognition, memory, visual perception (67), depression (68), and total cerebral brain, occipital and frontal lobar gray matter volumes (69).

At issue is whether the necessary elements of the *chronic* stress explanation (recurrent exposure, uncontrollability, and perceived social evaluative threats) were sufficiently present to inhibit recovery from elevated cortisol levels. Given that the initial dose and boosters at 5 and 11 months lasted 2–6 weeks and were completed less than a month prior to their respective data collection points, training exposure, especially as it relates to outcome assessment, could be viewed as recurrent. Because the visual speed of processing intervention was adaptive, its participants could have felt that they had less control over their intervention, especially because crossword puzzles were a common pastime for older adults while visual speed of processing training was an entirely new experience for most of them. These recurrent exposures of a novel, uncontrollable, and challenging experience may have led to negative self-identity perceptions (i.e., perceived social evaluative threats) and greater frustration among the participants in the visual speed of processing group. Thus, the presence of the essential elements of the chronic stress model may have been sufficiently present to support the *chronic* stress explanation.

That said, two additional issues need to be addressed. The first is that we did not measure cortisol levels. Therefore, we cannot demonstrate the hypothesized etiologic mechanism for the *chronic* stress explanation of inhibited recovery from elevated cortisol levels. Cortisol levels were not measured because the goal of our RCT was simply to see if the results from prior studies of healthy community-dwelling older adults (16–19,51,52) could be extended to assisted and independent living communities. The second issue is why the *chronic* stress explanation would only apply to participants in the visual speed of processing training intervention in assisted living communities. As noted above and in Table 1, there are important differences in age, self-rated health, comorbidity, and speed of processing between those in the assisted versus independent living communities. All of these differences favor those in independent living. If there were maximal thresholds on these characteristics above which the intervention would not be effective, then these differences could result in visual speed of processing training being less effective, or even harmful for those in assisted living communities.

### Potentially Plausible Methodological Explanations

There are also several methodological issues that might account for our findings. The visual speed of processing training used in the MOOD Study involved second-generation, gamified Windows platforms versus the first-generation MS-DOS platform used in ACTIVE (16–19). Moreover, individual rather than group-based (three to five participants) training was used in the MOOD Study, there was no standby trainer, no discussion of the merits of the cognitive training for everyday activities after each session, no acceptability or cognitive assessment data were collected, the standardization and certification of the Study Liaisons was shorter and less formalized, and the crossword puzzle program that the attention control group used was not adaptive and therefore the potential adaptive effect is masked by the visual speed of processing training effect. Finally, only 31 senior living communities from one rural state were included and follow-up was just 1 year, limiting generalizability and prohibiting the assessment of long-term effects.

The importance of some of these methodological differences, however, may be tempered by several factors. The compact disk version (*Road Tour*) of the visual speed of processing training was previously used in the Iowa Healthy and Active Minds Study (52) which had three intervention groups—onsite laboratory-based training with and without boosters, and at-home training. Positive results (Cohen’s *d*_*s*_ of −0.32 to −0.58) on the Useful Field of View test (37) were observed for each of those intervention groups. In addition, acceptability data from that community-based study were high. Moreover, while the crossword puzzle attention control group was not adaptive, it was more rigorous than the no-contact control group used in ACTIVE (16–19), perhaps accounting for the somewhat smaller effect sizes reported in the Iowa Healthy and Active Minds Study (52). Furthermore, while *Road Tour* did have a gaming element, it was removed from the *Double Decision* platform, with both having equivalent effects (28). Finally, although the MOOD Study failed to include cognitive assessments, the sensitivity analysis restricted to those with Useful Field of View (37) scores faster than the mean for the independent living communities suggested that our results were robust.

### Where Do We Go From Here?

First, there is the obvious need for further research to see if the harmful effects of visual speed of processing training reported here are replicable and generalizable beyond the two modalities (*Road Tour and Double Decision*) used in the MOOD Study. These new research studies must have larger samples that are more representative of the nation, target community-dwelling as well as assisted and independent living communities, use enhanced data collection techniques that include a full and repeated complement of cognitive and neuropsychological testing as well as acceptability assessments, track participants over the long term, use fully adaptive crossword puzzle and/or other computerized game attention control groups, include alternative (nonvisual) processing speed training platforms, and have sequential stopping rules for efficacy, harm, and futility. Finally, the collection of data on potential moderators and etiological mechanisms, as well as biomarkers and neuroimaging, are needed. Specifying those potential moderators and etiological mechanisms, however, is problematic because to our knowledge there are no other theoretical or evidentiary bases that provide guidance for the harmful heterogeneity of treatment effect among those in the assisted living communities.

Second, there is the question about new or continued use of visual speed of processing training in assisted living communities. Should the unexpected effect from a single, moderate-sized, short-term follow-up RCT (the MOOD Study) result in a moratorium or even a cautionary call against using visual speed of processing training in assisted living communities? On the one hand, there was a medium-sized statistically and clinically important harmful effect that was robust to several sensitivity analyses. On the other hand, and as noted above, the MOOD Study had a number of limitations that make its results less than definitive. Under these circumstances, it would seem most appropriate to sound a cautionary warning about the use of visual speed of processing training in assisted living communities until results from the additional research outlined above become available in the scientific literature. But such a cautionary warning should only apply to the two modalities of visual speed of processing training *(Road Tour* and *Double Decision*) used in the MOOD Study. This is because there is simply no evidence of harmful heterogeneity of treatment effects for those in assisted living from any other visual speed of processing training interventions, or for that matter, from any cognitive training interventions that do not rely on visual speed of processing (like the episodic memory or inductive reasoning interventions used in ACTIVE) (16–19).

Finally, there is the question of what if any aspects of the two modalities of visual speed of processing training (*Road Tour *and *Double Decision*) used in the MOOD Study might account for the harmful effects on health-related quality of life in the assisted living communities. Although our study cannot provide data to directly address this question, some speculation seems warranted, especially in the context of comparisons to the attention control group. First, as noted above, the crossword puzzles intervention that the attention control group faced was not adaptive, and therefore its participants did not face progressively more complex puzzles after reaching a success threshold like the 75% correct level for those in the visual speed of processing intervention. Second, crossword puzzles are a common and enjoyable pastime for many older adults, and the switch to solving them on a computer rather than on paper was likely not much of a challenge for those in the attention control group. In contrast, the visual speed of processing training was quite likely something that the majority of older adults had never before experienced. Third, in the attention control group the crossword puzzles changed after each puzzle was completed or the participant chose to move to another one. In contrast, those in the visual speed of processing intervention experienced rather slow and relatively minor changes in what they viewed. Finally, combined with the help functions that allowed the participant to let the computer fill in letters or words, show incorrect entries, and/or solve the entire puzzle, those in the attention control group may have felt more in control of their intervention than those in visual speed of processing intervention, as well as less frustrated.

## Supplementary Material

igaa029_suppl_Supplementary_Material
