# Supplementary material for: Does Visual Speed of Processing Training Improve Health-Related Quality of Life in Assisted and Independent Living Communities?: A Randomized Controlled Trial
Source: Innov Aging. 2020 Jul 31;4(4):igaa029. doi: 10.1093/geroni/igaa029 (PMC7489078; doi:10.1093/geroni/igaa029)
Supplement: igaa029_suppl_Supplementary_Material [file igaa029_suppl_supplementary_material.docx]

Online Supplementary Material

Supplemental Table 1. The psychometric properties from exploratory factor and reliability analyses of the covariate summary scales.

| Covariate Summary Scale | Number of Items | Simple Factor Structure | Percent of Variance Explained | Minimum Factor Loading | Coefficient Alpha |
| --- | --- | --- | --- | --- | --- |
| Useful Field of View | 3 | Yes | 71.7% | 0.58 | 0.77 |
| Depression (PHQ-9) | 9 | No* | 49.2% | 0.53 | 0.73 |
| Anxiety (GAD-7) | 7 | Yes | 63.7% | 0.45 | 0.82 |
| Social Support (MOS) | 5 | Yes | 59.2% | 0.75 | 0.83 |

*The depression scale had one main factor, and one minor factor (eigenvalue = 1.05).

Supplemental Table 2. The estimated marginal means on the Mental Component T-Scores for the Assisted Living and Independent Living Communities, by Visual Speed of Processing Training vs. Attention Control groups, obtained from the four progressive intention-to-treat, random effects linear mixed models including the interaction term for Visual Speed of Processing Training and Assisted Living.

| Models (M_n_) | Assisted Living Communities | | Independent Living Communities | |
| --- | --- | --- | --- | --- |
|  | Visual Speed of Processing Training | Attention Control | Visual Speed of Processing Training | Attention Control |
| M_4:_  Equivalence Expectation, Clustering, Visual Speed of Processing Training, Assisted Living, and Assisted Living Interaction Effects | 46.7 | 50.3 | 51.2 | 49.4 |
| M_5:_  M_4_ + Baseline Age, Sex, Education, and Comorbidity Main Effects | 46.1 | 49.9 | 51.5 | 49.8 |
| M_6:_  M_5_ + Baseline Useful Field of View Composite Score Main Effect | 46.4 | 50.7 | 51.5 | 49.4 |
| M_7:_  M_6_ + Baseline Depression, Anxiety, and Social Support Scores | 46.7 | 50.9 | 51.3 | 49.1 |

Supplemental Figure 1. CONSORT flow chart for the MOOD Study.

Loss to follow-up (n = 25)

Number analyzed (n = 148)

Mean treatment dose = 9.8 hours,

*IQR* = 4.9 to 14.7 hours

Loss to follow-up (n = 26)

Number analyzed (n = 152)

Mean attention control dose was not

captured for crossword puzzles

Randomized (n =351)

Allocated to the visual speed of processing training treatment group

(n = 173)

Mean treatment dose = 8.9 hours,

IQR = 3.3 to 13.7 hours

Allocated to the attention control group

(n = 178)

Mean attention control dose was not

captured for crossword puzzles

Did not meet inclusion criteria (n = 19)

Assessed for eligibility (n = 370)

**Enrollment**

**Allocation**

**Twelve Months**
